# Modeling of Blood Flow Dynamics in Rat Somatosensory Cortex

**DOI:** 10.3390/biomedicines13010072

**Published:** 2024-12-31

**Authors:** Stéphanie Battini, Nicola Cantarutti, Christos Kotsalos, Yann Roussel, Alessandro Cattabiani, Alexis Arnaudon, Cyrille Favreau, Stefano Antonel, Henry Markram, Daniel Keller

**Affiliations:** Blue Brain Project, École Polytechnique Fédérale de Lausanne (EPFL), Campus Biotech, 1202 Geneva, Switzerland

**Keywords:** blood flow, astrocytic endfoot, vasodilation, neuro-glia-vasculature, simulation

## Abstract

**Background:** The cerebral microvasculature forms a dense network of interconnected blood vessels where flow is modulated partly by astrocytes. Increased neuronal activity stimulates astrocytes to release vasoactive substances at the endfeet, altering the diameters of connected vessels. **Methods:** Our study simulated the coupling between blood flow variations and vessel diameter changes driven by astrocytic activity in the rat somatosensory cortex. We developed a framework with three key components: coupling between the vasculature and synthesized astrocytic morphologies, a fluid dynamics model to compute flow in each vascular segment, and a stochastic process replicating the effect of astrocytic endfeet on vessel radii. **Results:** The model was validated against experimental flow values from the literature across cortical depths. We found that local vasodilation from astrocyte activity increased blood flow, especially in capillaries, exhibiting a layer-specific response in deeper cortical layers. Additionally, the highest blood flow variability occurred in capillaries, emphasizing their role in cerebral perfusion regulation. We discovered that astrocytic activity impacted blood flow dynamics in a localized, clustered manner, with most vascular segments influenced by two to three neighboring endfeet. **Conclusions:** These insights enhance our understanding of neurovascular coupling and guide future research on blood flow-related diseases.

## 1. Introduction

The interrelationships among astrocytes, neurons, and blood vessels are particularly complex, giving rise to a system of multidirectional communication, the neuro-glia-vascular (NGV) ensemble [1]. Astrocytes play an important role in it and in energy metabolism [2]. Of particular note are the specialized astrocytic processes known as endfeet, which establish direct contact with arterioles, venules, and capillaries, securely anchoring them to the vascular surface [3,4,5]. Several investigations based on chemical fixation for tissue preservation have reported that astrocytic endfeet cover at least 60% of the total surface area of blood vessels [3,5,6,7]. In accordance to that, we build upon the recent in silico NGV circuit developed by Zisis et al. [8] that exhibits a vascular coverage of approximately 60%.

Astrocytes regulate some aspects of blood flow and the exchange of molecules to and from the brain. This regulation is partially coordinated by neuronal activity monitored by astrocytes and mediated via their endfeet. The release of substances from interneurons, such as nitric oxide, can also influence vasodilation. However, this is outside the scope of the current study [9,10]. In this work, we did not explicitly model the mechanisms that generate vasomodulatory signals in the endfeet. Instead, we approximated their effect on vessel radii using an Ornstein–Uhlenbeck (OU) process.

The exchange of molecules, especially the delivery of essential nutrients, predominantly occurs at the capillary level due to the proximity of the capillaries to tissue. Capillary blood flow increases during neuronal activation. Consequently, alterations in the diameters of blood vessels, especially at the capillary level, significantly modify blood flow in the vascular network. In turn, these changes impact the distribution of vital nutrients essential for the support of neuronal metabolism, including lactate, glucose, and oxygen [11,12,13].

The astrocytic response shows a noticeable delay of several seconds after neuronal activation, as expected from the neuronal release of neurotransmitters that initiate astrocytic activation. This response is transmitted to neighboring astrocytes, causing alterations in the perfusion of nearby capillaries. These changes in perfusion may be the result of vasodilation or vasoconstriction in the capillaries [14,15,16,17].

Hemodynamic simulations commonly employ mathematical models of cerebral microcirculation that combine biophysical principles with medical imaging data, including X-ray tomographic microscopy [18]. These models integrate anatomical features such as branching patterns and vessel density with hemodynamic and metabolic processes. They provide insights into fundamental anatomical principles, geometric constraints (such as spatial organization, dimensions, and architecture of blood vessels), predictions of cerebral blood flow dynamics, and refined characterizations of metabolite exchange between tissues [19,20,21,22,23,24,25]. In addition, established microcirculatory assessment methods, such as laser Doppler flowmetry and capillaroscopy, have laid the foundation for the analysis of vascular function and flow heterogeneity [26,27] providing context for our modeling approach. In hemodynamic simulations, critical anatomical aspects include the organization of cerebral microcirculation and structural characteristics such as the arrangement of blood vessels, tissue composition, and spatial relationships between different anatomical elements. Geometric constraints are imposed by the physical geometry of the microvasculature network, including the size, shape, and branching patterns of blood vessels [28,29]. For instance, narrow or tortuous vessels can increase resistance to flow, thereby influencing overall hemodynamics and affecting the distribution of blood and nutrients in different regions of the brain [30,31]. Astrocytic endfeets can modulate blood flow [32,33,34,35,36,37] by regulating vascular diameter [38,39].

In this paper, we investigate how changes in vessel diameters alter blood flow within a complex vascular network. This study explores the dynamic nature of blood circulation within a complex network of blood vessels, demonstrating how variations in vessel diameter influence overall flow patterns. Furthermore, we validated the framework against experimental observations regarding flow values across cortical depths (from cortical layer 1 to layer 6, L1–L6). By employing a multiscale in silico model, we integrated the role of astrocytes in the regulation of cerebral blood flow, a crucial factor in vasodilation and vasoconstriction [33]. Our approach provides a foundation for the interpretation of depth-dependent flow measurements, offering insights that are difficult to obtain through experimental methods alone. This integration highlights the significant contribution of astrocytes to blood flow regulation within the neurovascular unit, thus establishing a comprehensive framework to understand cerebrovascular dynamics [40].

## 2. Materials and Methods

Our simulation framework consists of three primary components:

- Coupling between microvasculature and astrocytic morphologies. This component ensures that the microvasculature is intricately connected with the synthesized morphologies of astrocytes throughout the circuit.
- Fluid dynamics model. This model calculates the flow and pressure within each segment of the vasculature, providing detailed information on hemodynamic behavior.
- Stochastic radii simulation. We used a reflected Ornstein–Uhlenbeck (ROU) process to simulate the dynamic changes in the radii of the vessel, capturing the stochastic nature of vascular adjustments.

Several studies have reported that vasoconstriction is significantly less pronounced than vasodilation in all types of vessels [14,41,42,43,44,45]. Thus, we chose to focus solely on vasodilation for the scope of this paper for several reasons. First, vasodilation generally results in larger-amplitude changes in the vessel diameter compared to vasoconstriction (as for the pial vessels, the amplitude of constriction is significantly lower than for dilation). This makes it easier to detect and measure the effects accurately, providing more reliable data for the model. Second, vasodilation plays a critical role in many physiological and pathological conditions, especially in response to increased metabolic demand and during certain diseases. Therefore, focusing solely on vasodilation enables a more direct examination of these relevant conditions. Lastly, by focusing on only one mechanism, we can simplify the model, reducing computational complexity, and allowing for a more targeted analysis.

Algorithm in Section 2.6 also presents a summary of the algorithm for simulating blood flow modulation driven by astrocytic activity.

### 2.1. Digital Cerebral Microvasculature Network

**Figure 1:**
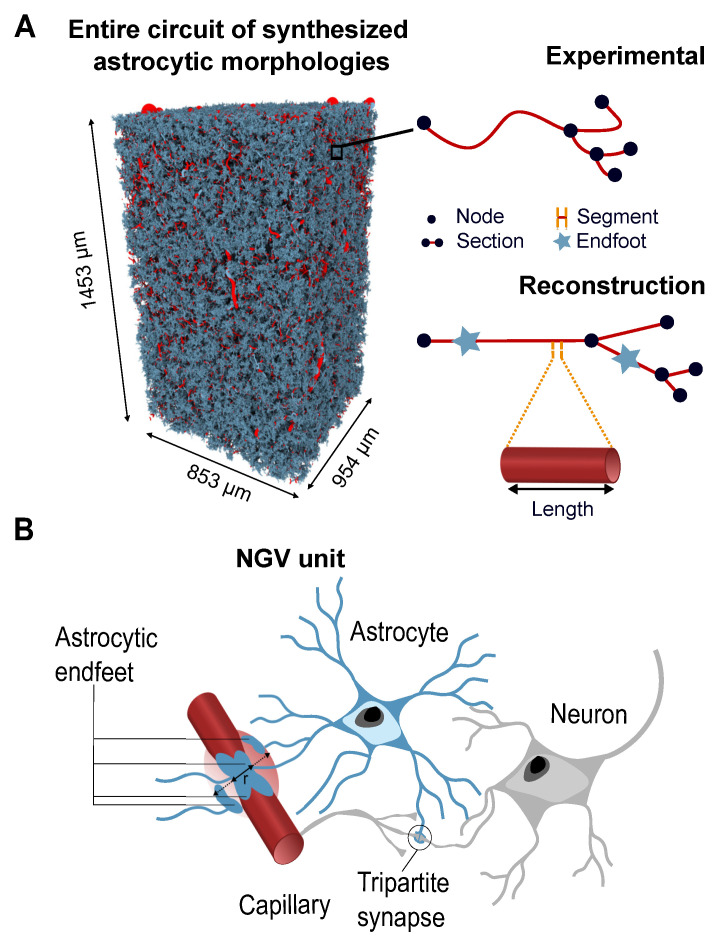
Model overview. (**A**) The left side shows a complete circuit with synthesized astrocytic morphologies developed by Zisis et al. [8]. Astrocytes are shown in blue and blood vessels in red. On the right, there is a schematic depiction of a realistic microvascular network sample, comprised of nodes and edges. A dot represents a node, two dots connected by a line represent an Section (an edge between every bifurcation), a black line framed by vertical orange lines represents a Segment (an edge between every Section), and a star represents an endfoot. (**B**) NGV unit. Neurons are depicted in gray, astrocytes in blue, and capillaries in red. Astrocytes contact synapses, wrap around them, and extend their perivascular projections to the surface of blood vessels, where they form endfeet. *r* stands for radius.

The cerebral microvasculature is an intricate network of interconnected blood vessels [46]. For this study, we used the NGV digital reconstruction of the rat somatosensory cortex (SSCx) presented in [8]. The dimensions of this circuit (954 μm × 1453 μm × 853 μm) were determined by colocalization of the vascular data set [8,18] with a neuronal mesocircuit [47]. These dimensions equate to a volume of 1.18 mm^3^, which accounts for approximately 0.2% of the rat brain volume. A total of 14,402 astrocytes populate the bounding region, each with two endfeet, resulting in a total count of 28,804 endfeet [8] (see Figure 1A, where the astrocytes are blue and blood vessels are red).

The vasculature dataset is represented as a graph composed of nodes and edges. Each node is characterized by its coordinate position and radius, while the edges indicate the connections between the nodes. The length of an edge is simply the distance between the connected nodes, and the edge radius is defined as the arithmetic average between the radii of the nodes. The microvascular network (see Figure 1) serves as the structural framework for the placement of astrocytic endfeet.

In a realistic network, each edge corresponds to a blood vessel and is located between two bifurcations (branching nodes) and typically exhibits tortuosity. In the model, we simplified the reconstruction of the network by disregarding the tortuosity while still accounting for the actual lengths of the tortuous vessels. As a result, each edge was modeled as a straight pipe with a fixed radius. Figure 1A illustrates a representation of a cerebral microvascular network.

**Figure A5:**
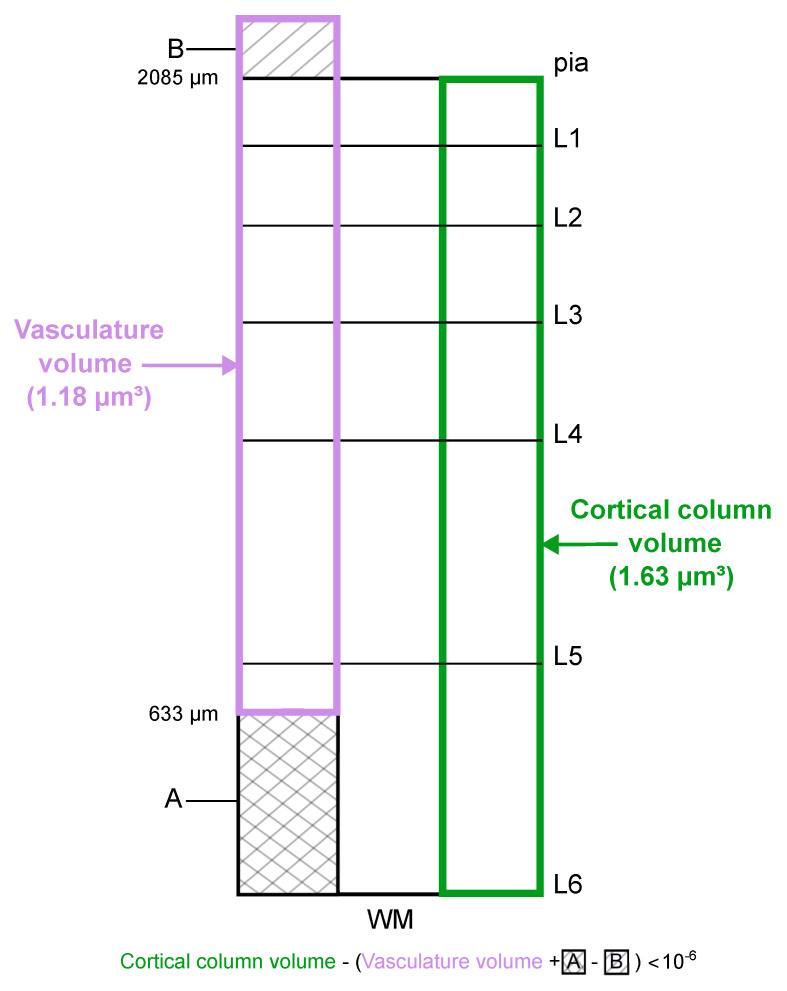
Volume discrepancy: the misalignment between the vasculature and microcircuit in the rat SSCx. The pink rectangle represents the vasculature volume, while the green one shows the cortical column volume. The (B) striped volume represents the volume of the vasculature above L1, while the (A) hatched volume represents the missing vasculature from three-fourths of L6 down to the white matter (WM).

The volume of the vasculature was extracted from rat SSCx [18]. Then it was aligned with the cortical column microcircuit using cortical layer boundaries as the neuronal region in rat SSCx is usually subdivided into six layers [48]. A significant section of the vasculature is located above L1 and should be taken into account as it contains the main arteries. Moreover, the current vasculature volume [18] is truncated approximately one-fourth of the height of L6, from the top. Therefore, three-fourths of L6 down to the white matter have no vasculature in this model. Thus, the vasculature and microcircuit volumes do not completely overlap (see Figure A5).

Table 1 provides data for several quantities inside the six-layered structure, such as the volume of the layer, the vascular volume, the proportion of the total volume occupied by blood vessels, and the proportion of flow passing through capillaries and large vessels.

The low percentage of vessels within L6 can be attributed to the limitations of the experimental data set. This data set only contained a cropped portion of L6, resulting in a relatively small representation of this layer. It was imperative to consider L6 while performing our analyzes, even if its data set was limited. The flow ratio highlights the proportion of flow in capillaries and large vessels. The sum was smaller than 100% as we exclusively considered capillaries (diameters smaller than 6 μm) and large vessels (diameters larger than 14 μm) among various types of vessels. The decision to exclude other vessels was made to emphasize the distinctive behaviors of capillaries and large vessels. Capillaries are crucial for the exchange of nutrients and gases at the cellular level, while large vessels are vital for the rapid transport of blood and the regulation of pressure. By focusing on these extremes, our analysis could better capture the specific roles and responses of these vessel types under various physiological and pathological conditions. This approach facilitated easier validation of our results against the existing literature because extensive research and data are more readily available for these types of vessels, providing well-documented benchmarks for comparison [49,50,51].

### 2.2. Mathematical Framework of Blood Flow Model

We present the mathematical framework of the fluid dynamics model that characterizes the flow and pressure of blood within the vascular system. The vasculature is modeled as an intricate graph of interconnected blood vessels. In turn, each one of them is represented as a tube of length *l* and with circular cross section $A\left(t\right)=\pi \;r{\left(t\right)}^{2}$, where r(t) is the radius and *t* is time. The vessel section A(t) can change with time due to the effect of the endfeet and is constant along the longitudinal axis. The blood is treated as an incompressible and viscous fluid that circulates within each vessel. The velocity of blood u(t,x) and the pressure p(t,x) are assumed to be constant in the radial direction. Therefore, they only depend on the longitudinal spatial dimension *x*, and  the entire system can be considered one-dimensional.

The mathematical framework considers the following 1D Navier–Stokes momentum equation:

(1) $$\frac{\partial u(t,x)}{\partial t}+u\left(t,x\right)\;\frac{\partial u(t,x)}{\partial x}+\frac{1}{\tilde{r}ho}\frac{\partial p(t,x)}{\partial x}=-\frac{R(r)}{\tilde{\rho }}u\left(t,x\right),$$

Here, $\tilde{\rho }$ is the constant fluid density and R(r) is the resistance with the following expression:

(2) $$R\left(r\right)=4\;R^{Pois}\left(r\right)\;\;\left(1-\alpha \;e^{-r/\beta }+\gamma \;e^{-r/\delta }\right).$$

$R^{Pois}\left(r\right)$ is the classical Poiseuille resistance, defined as follows:

(3) $$R^{Pois}\left(r\right)=\frac{8\eta }{r^{2}}\;\left(\;\mathrm{or}\;\mathrm{alternatively}\;R^{Pois}\left(A\right)=\frac{8\eta \pi }{A}\;\right).$$

The radius is expressed in (μm) while η represents the viscosity of the plasma ($1.2\times 10^{-6}\;g\cdot \mu m^{-1}\cdot s^{-1}$). In this study, the blood flow is modeled by assuming a constant plasma viscosity. Although it is well established that blood exhibits non-Newtonian behavior, with viscosity that varies as a function of shear rate and hematocrit concentration, this simplification allows for a more tractable analysis of vascular flow dynamics, particularly in complex networks. Previous studies have shown that such an approach can still produce reasonably accurate insights when the main focus is on comparative or qualitative trends rather than precise rheological detail [52].

We developed a matrix-based formalism to solve the equations in the vascular network in each time step, deriving the flow from the pressure. The details of our approach are provided in Appendix A.1.

### 2.3. Blood Flow in the Boundary Nodes

The static flow model described in Section 2.2 and Appendix A.1 computes the flow along each edge of the graph, given the flows assigned to the boundary nodes. The simulation time [0,T] was discretized in I time steps and I+1 time points. We solved Equations (A7) and (A8) for each time point with different boundary conditions and different values of the vessel radii. The present section focuses on the time-dependent boundary conditions $Q^{b}\left(t\right)$ for t∈[0,T], while Appendix A.3 shows how radii dynamics are modeled.

Our approach has three main components:

1. Entry node selection. The nodes where blood is introduced into the system.
2. The formulation of a time-dependent flow model. It is necessary to characterize the flow injected within the entry nodes.
3. Exit flow calculation. Calculation of the quantity of blood that leaves the vasculature through the exit nodes.

The details of our approach are described in Appendix A.2.

### 2.4. Stochastic Endfeet Activity

As explained in Section 2, our study focuses exclusively on vasodilation. We decided to employ a reflected Ornstein–Uhlenbeck (ROU) process [53,54] to replicate the dynamics of the radius of a vessel due to vasodilation. It is a stochastic process that always reverses to the resting state (RS). The reason we used the reflecting boundary for the RS is because the radius can never go below the RS; this is the reflection level. This process is both reverting and reflecting, reverting to the RS and reflecting because it goes back up where it arrives at the RS. It does not go down. It does not go below the RS Consequently, we used an ROU process with a zero reflection barrier to prevent negative values, thereby accurately replicating the vessel radii dynamics influenced by endfeet activity.

The details of our approach are described in Appendix A.3.

### 2.5. Passive Response of Network

In our model, the phase corresponding to the passive response of the vasculature corresponds to the phase when there is no astrocytic activity (i.e., no noise). We can remove the Wiener noise in Equation (A14) in Appendix A.3 by setting σ=0.

Let us define τ as the time at which end-foot activity ceases. During the passive response phase, for t>τ, the radius dynamics decays exponentially as follows:

(4) $$r_{t}=r_{0}+e^{-\kappa (t-\tau )}\;\left(r_{\tau }-r_{0}\right)\;\mathrm{for}\;t>\tau .$$

The average radius dynamics among all edges, ${\bar{r}}_{t}$, can be determined through the following:

(5) $${\bar{r}}_{t}={\bar{r}}_{0}+e^{-\kappa (t-\tau )}\left({\bar{r}}_{\tau }-{\bar{r}}_{0}\right)\;\mathrm{for}\;t>\tau .$$

Here, ${\bar{r}}_{0}$ and ${\bar{r}}_{\tau }$ represent the average blood vessel radius at the times 0 and τ, respectively.

### 2.6. Algorithm Implementation

In the previous sections, we introduced a mathematical model for computing the flow and pressure within a vasculature in the presence of astrocytic activity. The Algorithm 1 outlines all the necessary steps for the simulation.

**Algorithm 1:** Simulation of blood pressure and flow during astrocytic stimulation.

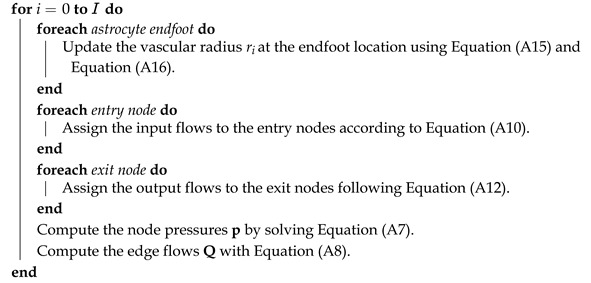



The algorithm, implemented in Python, was released as an open-source package named AstroVascPy (version 0.1.6; Battini, S et al., Geneva, Switzerland). The source code is available for public access on GitHub [55]. The software was designed for scalability across diverse vascular network datasets, with the possibility to use parallel computation with modern HPC clusters.

The part of the code that required the most computational resources is the solution of the sparse linear system Equation (A7). After several tests, we found that for graphs with ∼$10^{6}$ nodes, it is convenient to solve the system with a direct method, while for very large graphs (more than $10^{7}$ nodes), it is more efficient to use an iterative parallelized algorithm. For this purpose, we relied on the Python package petsc4py (version 3.22.2) [56], or PETSc python binding, a well known C library for numerical parallel computation.

The particular vasculature network considered in this paper had *N* = 1,351,448 nodes and *M* = 1,349,411 edges. In our analyses, we reduced the graph to its largest connected components. We then obtained *N* = 1,049,221 and *M* = 1,051,911. The Laplacian *L* in Equation (A7) is a sparse N×N matrix. In this case, the system was quite small and could be solved quickly by a direct method such as the LU decomposition. We relied on the Python library *scipy.sparse* for this computation.

### 2.7. Mathematical Framework for Quantitative Analysis

In this section, we define the key quantities that will be essential for analyzing the results in Section 3. In each time step, *i*, and at each edge, *k*, we denote the flow as Q(i,k) and the radius as r(i,k). For the edges connected to the endfeet, the quantities are denoted with the superscript *e*, e.g., $Q^{e}\left(i,k\right)$, $r^{e}\left(i,k\right)$, etc.

We define the *resting state ratio* for the flow and the radius as follows:

(6) $$RSQ_{i,k}=\frac{Q(i,k)}{Q(0,k)}\;\mathrm{and}\;RSr_{i,k}=\frac{r(i,k)}{r(0,k)}\;\;\forall \;k,i>0$$

Consider a subset of the set of all edges that contains $M^{\prime }$ edges, with $M^{\prime }\leq M$. We define the *average ratio* for the flow over a selected subset of edges as follows:

(7) $$ARQ_{i}\;=\;{\langle RSQ_{i,k}\rangle }_{k}\;=\;\frac{1}{M^{\prime }}\sum_{k=0}^{M^{\prime }}RSQ_{i,k}\;\;\forall \;i\geq 0$$

We use brackets 〈〉 to indicate an arithmetic average with respect to the variable indicated in the subscript.

When we take an average over a set of edges and over time, we denote it as

(8) $$ARQ\;=\;{\langle ARQ_{i}\rangle }_{i}\;.$$

#### Order Ratios

The distance order *m* of an edge $\tilde{k}$ with respect to the target edge *k* is defined as the minimum number of connected edges that we need to traverse to reach $\tilde{k}$ starting from *k*. For example, if two edges share a node, their distance order is 1. If we need to pass through an extra node to move from the initial edge to the final edge, the distance order is 2, and so on.

Now, let us introduce the concept of the *order ratio* for the flow to quantify how the astrocytic activity on an edge, *k*, affects segments in the surrounding vascular network. We define it as follows:

(9) $$ORQ_{m}=\;{\langle \;{\langle \;{\langle \;\frac{RSQ_{i,\tilde{k}}}{RSQ_{i,k}^{e}}\;\rangle }_{\tilde{k}}\;\rangle }_{k}^{e}\;\rangle }_{n},\;\mathrm{for}\;\tilde{k}\in K_{m}\left(k\right)$$

where $K_{m}\left(k\right)$ is the set of all edges of distance order, *m*, from a target edge, *k*. The most interior average was calculated over the elements of $K_{m}\left(k\right)$. The average in the middle was calculated over all edges, *k*, connected to endfeet. And, finally, the last average was calculated over all time points, *n*.

## 3. Results

In this paper, our main goal was to characterize the interactions between astrocytic activity and blood flow within a large and complex vascular network. To achieve this, we developed a model that simulates the effect of astrocytes on vessel radii using a reflected Ornstein–Uhlenbeck process (see Section 2.4 and Appendix A.3). Subsequently, we used the Hagen–Poiseuille equation, which is derived from the 1D Navier–Stokes momentum equation to calculate the blood flow in each segment of a given vascular network (see Section 2.2 and Appendix A.1). We decided to apply this model to Reichold et al. rat SSCx vasculature [18] since the placement of astrocytes has previously been performed in this vasculature [8].

### 3.1. Model Presentation

**Figure 2:**
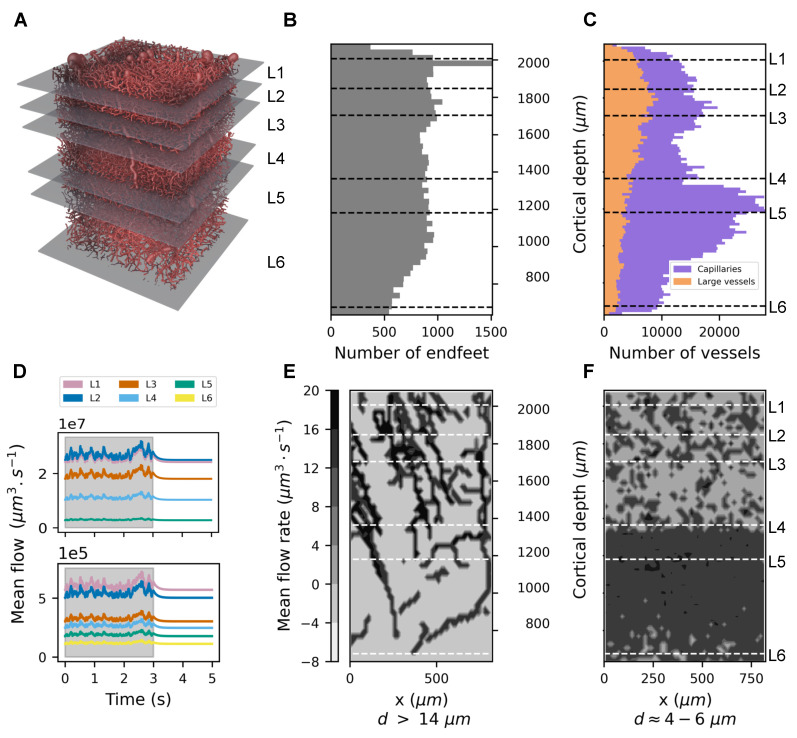
Model presentation. (**A**) The vasculature segmented into six cortical layers in the rat SSCx separated by gray planes. (**B**) The endfeet distribution along the cortical depth in the vasculature [8]. (**C**) The spatial distribution of capillaries and large vessels along the cortical depth [8]. (**D**) A time series illustrating the flow variations averaged across all large vessels (top) and capillaries (bottom) in each cortical layer (color-coded) in response to a three-second astrocytic stimulation (gray rectangle). Since there are no large vessels in L6, a corresponding time series is missing from the legend. (**E**) HA heatmap of the mean flow in the vasculature at a specific time (t=3s) within the simulation, calculated as an average over a surface area of 17 μm× 21 μm within large vessels (diameter d≥14μm). (**F**) A heatmap of the mean flow in the vasculature at a specific time (t=3s) within the simulation, calculated as an average over a surface area of 17 μm× 21 μm within capillaries (diameter d≈4–6μm). The vertical axis in (**B**,**C**,**E**,**F**) represents the cortical depth.

Throughout this paper, we focus our analyses on the six cortical layers of the rat SSCx due to their distinct functional and structural characteristics (see Figure 2A). Understanding the organization and dynamics of these layers is crucial for capturing the complex neural processes underlying sensory perception and integration [57,58,59]. Traditional vascular studies typically categorize vessels into two types: capillaries with a diameter d∈[4–6]μm and larger vessels (d≥14μm). Thus, we used a similar categorization to study the interaction between endfeet activity and the vascular network. The astrocytic endfeet distribution was quite constant between cortical layers one to four with around 800 endfeets per bin of 25 μm along the cortical depth (Figure 2B). The number of endfeet then decreased within L5 from 800 to 500 endfeet per 25 μm bins. On the other hand, we observed a prevalence of capillaries within the deeper layers (L4–L5), while large vessels were prevalent in the upper layers (L1–L2); see Figure 2C. As expected, the number of large vessels decreased in the deeper layers. The incompleteness of the vasculature data set did not allow us to observe the endfeet distribution in L6.

A typical simulation consisted of a three-second long injection of flow into the vasculature entry nodes (Figure 2D, gray rectangle), accompanied by a stochastic expansion of the radii of the vasculature sections connected to the endfeet (see Section 2). For the sake of simplicity, in the following analyses, we use a constant blood velocity of 3.5 $\times 10^{4}\;\mu m\cdot s^{-1}$. This is equivalent to the set $\tilde{A}=0$ in Equation (A9). We then simulated the next two seconds without astrocytic activity to observe the response of the vasculature through a relaxation period (see Appendix D). Thus, we computed the flow and pressure variations for each time step in all segments of the vasculature during the time in which the astrocytes are active (from zero to 3 s) and the relaxation period (from 3 to 5 s). The results could then be easily classified according to various criteria, such as the cortical layers (L1 to L6) and the types of vessels (capillaries or large vessels). Figure 2D–F show typical simulation results. Figure 2D shows the time series of the mean flow rate grouped by layer and type of vessel (large vessels at the top and capillaries at the bottom). We observed, between the types of vessels and endfeet activity states, that the mean flow consistently exhibited higher values in the cortical layers L1 and L2, compared to the deeper layers (see Figure 2D). We observed very similar mean flow dynamics in both capillaries (Figure 2D, at the bottom) and the large vessels (Figure 2D, at the top). There was a two-order difference between the mean flow values of the capillaries and large vessels ($10^{5}$ and $10^{7}$, respectively). The mean blood flow dynamics in the capillaries (Figure 2D, at the bottom) tended to form two groups: L1 and L2 on one hand and L3–L6 on the other hand. This highlights the difference between the first two layers and the others regarding the quantity of the flow. Similarly, the mean flow in the large vessels (see Figure 2D, top) also showed a similar behavior in L1 and L2 and progressively decreased in the deeper layers.

Figure 2E,F depict heatmaps of the mean flow in the vasculature at a specific time (t=3s) for large vessels and capillaries, respectively. The cortical depth is represented by the y-axis, while the x-axis represents a second dimension of the vasculature. The values of the mean flow are then concatenated along the z-axis to create the heatmap representation. In the deeper cortical layers, there were more capillaries, resulting in more blood and increased total blood flow (Figure 2F). Conversely, the upper layers had more large vessels, leading to increased blood volume and higher total blood flow in that region (Figure 2E).

Overall, these results suggest that most of the variance of flow during the passive phase can be attributed to the architecture of the vasculature network. The distribution of blood flow is categorized into supragranular and subgranular layers and analyzed throughout this paper.

### 3.2. Astrocytic Activity Affects Flow in the Vascular Network

To characterize the model, we quantified the changes in vessel radii due to astrocytic activity and subsequent alterations in the blood flow.

**Figure 3:**
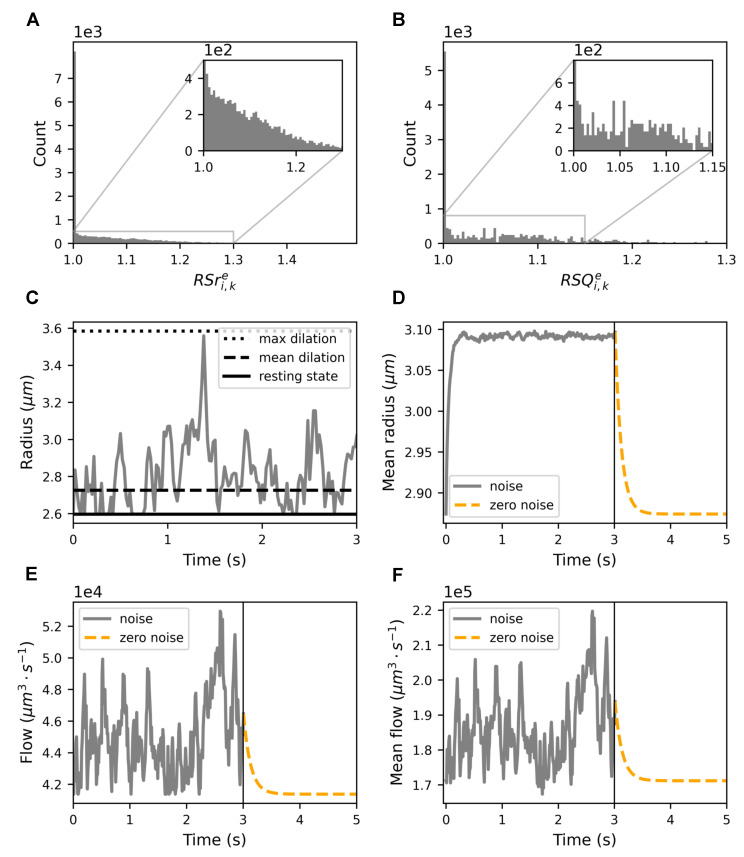
Dynamic analysis of blood vessel radii and flow in response to astrocytic activity. (**A**) Histograms showing the distribution of $RSr_{i,k}^{e}$ and (**B**) $RSQ_{i,k}^{e}$ for each edge connected to only one endfoot and for each time point (see Section 2.7). (**C**) A time series plot showing the radius dynamics for a single segment. The solid black line represents the resting state radius, the dashed black line represents the average radius over time, and the dotted black line indicates the theoretical maximal extension of the radius. (**D**) The time evolution of the mean radius across all blood vessels when the stimulation was halted after three seconds. (**E**,**F**) A presentation of the equivalent time series for the flow. (**D**–**F**) The solid gray line represents the stimulation period, while the dashed orange line illustrates the passive phase, after the stimulus ceased.

The responses to endfeet activity differ between capillaries and large vessels (radii modulation in Section 2). We used average data reported in the literature to set the maximum dilation, the *maximum radius ratio*, for both vessel types. In particular, it was set to 1.38 for capillaries and 1.23 for large vessels, based on [14,41,43,44,45,60,61,62]. We also set the time it took to reach the maximum radius, the *time to peak*, for both vessel types. The duration represents the average duration from the onset of the stimulus to the time when the maximum dilation was reached. In our simulations, we fixed this *time to peak* at 2.7 s for capillaries and 3.3 s for large vessels (see Section 2, Table 2 and Table 3, and [14,41,43,44,45,60,61,62]). Our choice of a 2.7-s time scale for the hemodynamic response represents an averaged simplification that captures the general dynamics observed experimentally. We acknowledge that fast (hundreds of milliseconds to seconds) responses are thought to be driven by rapid potassium signaling [63], while slower modulation occurs on the order of ten to hundreds of seconds, influenced by neuromodulators [64,65]. Nevertheless, the 2.7 s time scale was chosen to balance these effects and reflect intermediate responses typical in neurovascular modeling. In Figure 3A,B, we highlight the distribution of the resting state ratio for radius and flow. The histograms in Figure 3A,B consider all edges connected exclusively to a single endfoot, at every time point. According to the literature [14,41,43,44,45,60,61,62] and the implementation of our model (see Section 2), $RSr_{i,k}^{e}$ (see Equation (6)) was concentrated between 1 and 1.3, showing a peak close to 1.0 and gradually decreasing to 1.3.

Similarly, in Figure 3B, the distribution of the flow ratio of the resting state captures the temporal changes in the flow dynamics of the segments of the vessel connected to the endfeet. In other words, while in Figure 3A we explore variations in the input (i.e., the radii, $RSr_{i,k}^{e}$), in Figure 3B, we focus on the model output (i.e., the flow, $RSQ_{i,k}^{e}$). The area between 1 and 1.15 presents a high concentration of peaks of $RSr_{i,k}^{e}$, while the distribution remains relatively flat. The irregular shape of the flow distribution indicates a highly non-linear interaction between the radius dynamics (input) and the observed flow (output).

Such near-unity flow ratios indicate the predominant occurrence of minimal changes in the flow between successive time points. This observation underscores the robustness of the neurovascular system in maintaining cerebral blood flow within a narrow range, even in the presence of dynamic physiological processes such as neuronal activity and metabolic demands [66,67,68].

Figure 3C presents the path of the simulated dynamics for a single capillary taken in the center of the vasculature. The radius follows Equation (A16). The maximum dilation was obtained from data in the literature. Table 2 and Table 3 outline all the additional parameters used in the simulation.

Figure 3D shows the evolution of the mean radius during the simulation. Figure 3E presents the time series of the flow inside the capillary considered in Figure 3C.

Figure 3D–F presents the exponential decay profile of the mean radius, as predicted in Appendix D. According to Equation (4), the typical timescale for the vasculature to return to the resting state, the characteristic time, is approximately $\frac{1}{\kappa }$.

Figure 3D shows the mean of all radii during the simulated astrocytic stimulation over time. An increase in astrocytic activity led to a temporary increase in the mean value of the radii throughout the vasculature, indicative of an average expansion of the total vasculature volume.

Similarly, the equivalent plot for the flow in Figure 3E illustrates the response of a single blood vessel. Here, the increase in astrocytic activity leads to a transient increase in the flow.

We show in Figure 3F the time evolution of the mean flow across all blood vessels. As in the previous cases, it presents more noisy values. during the initial stimulation phase, and it then decreases exponentially in the following passive phase. The reason for a noisy path of the mean flow is due to the stochastic nature of the flow we injected into the vasculature. We kept the blood speed constant and multiplied it by the sectional area of the three entry nodes, which were all connected to the endfeet.

The similarity between the curves in Figure 3E,F could be explained by the principles of vascular physiology and the dynamics of NGV coupling, i.e., cerebral autoregulation and flow–metabolism coupling [69,70].

### 3.3. Simulation Results Reproduced Flow Ranges in the Literature

To validate the model, we compared its results with flow rates and velocities reported in the literature.

In our simulations, we imposed the flow value, $Q^{b}$, on the boundary nodes using Equation (A7). Thus, the validation focused solely on the blood vessels in the internal part of the vasculature, in the region where x∈[67.7,622.3]μm, y∈[1033.5,1684.9]μm, and z∈[273.27,926.73]μm. It produced 17% of the vasculature falling within the defined region. In this way, we minimized the possible distortions introduced by the boundary conditions and take into account the contributions of all nodes in the vasculature.

The simulation validation in Table 4 and Section 3.3 focuses on an analysis of flow values in capillaries categorized by their diameters.

In the literature, it is common practice to express flow rates in large vessels in nl/s and flow rates in capillaries in terms of the number of red blood cells (RBC) per second ($\mathrm{RBC}\cdot s^{-1}$). Table A3 recaps unit conversions and the parameters used in the simulations.

When interpreting flow values from the literature, it is crucial to consider potential selection biases and undetected artifacts. Biases may arise from the selective measurement of vessel segments, which might not be representative of the entire vascular network. Furthermore, blood flow measurements confined to specific regions or under specific conditions may not fully capture the variability present throughout the vasculature. In addition, artifacts can come from the resolution limits of instruments, leading to discrepancies between measured and actual values.

We classified capillaries into six categories, each distinguished by a diameter increase of 1μm (ranging from one to seven micrometers), to closely align with the existing literature [71]. Minimum, maximum, and mean values were recorded for each segment throughout the simulation. Next, we averaged these values on all segments in a particular diameter range. Table 4 shows the relationship between the capillary diameter and flow dynamics within the rat SSCx. Our analysis confirmed an increase in flow rates as the capillary diameter increased, with the highest flow observed in the capillaries within the 6–7 μm diameter range.

**Figure 4:**
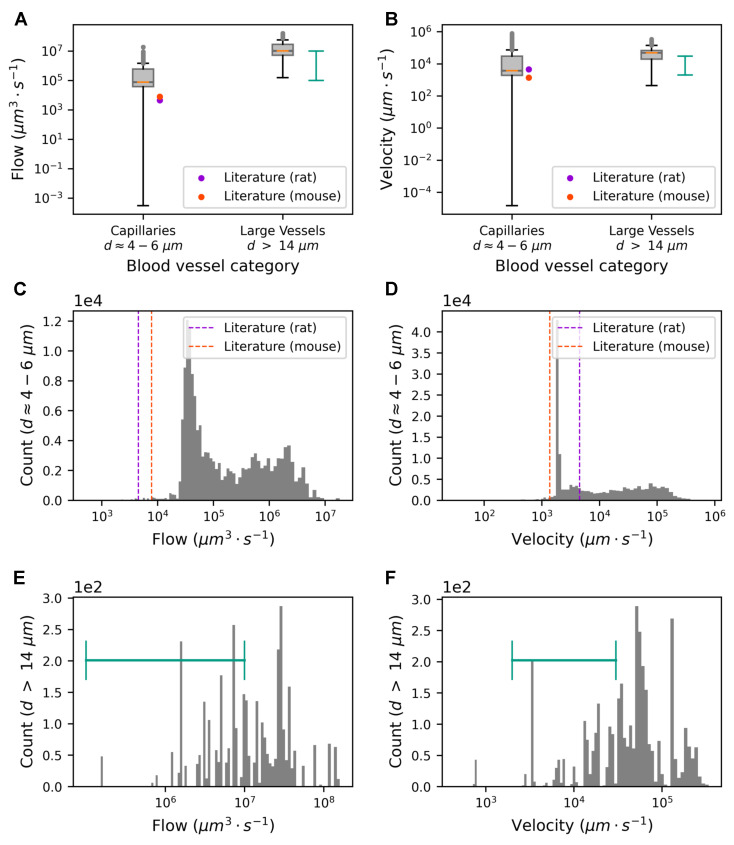
The validation of our simulation results against data in the existing literature. Blood flows (**A**) and velocities (**B**) were evaluated for both capillaries and large vessels. The mean values of the simulated flow/velocity were produced by averaging over all segments throughout the active astrocytic phase. Their distribution is shown in the boxplots. Gray dots show outliers, and orange lines indicate the median flow in blood vessels. Dot markers represent values from previous studies [73,74], color-coded by species. Error bars in turquoise–green depict the flow and velocity minimum and maximum observed for rats in [72]). The distribution of the blood flow (**C**) and velocity (**D**) in the capillaries (diameter d ≈4--6μm) plotted on a logarithmic scale. The dashed lines indicate values in the literature for rats and mice. The distribution of the blood flow (**E**) and velocity (**F**) in the large vessels (d≥14μm) plotted on a logarithmic scale. Error bars in turquoise–green depict the flow and velocity ranges observed for rats in [72].

The flows and velocities produced by the model align with the data in the literature for both capillaries and large vessels. In cases where average data were missing in the literature, only a range of values was reported from Shih et al. [72]. The flow and velocity ranges of the model were wider than the ones from the literature (see Figure 4A,B). It should be noted that our validation included data from thousands of vessels, a scale larger than in the typical literature, where data often come from only tens or hundreds of segmented vessels. Therefore, it is expected that our results show a wider range. Nevertheless, the bulk part of the model data (the second and third quartiles of the box plots) was in the typical range of the literature, close to $10^{4}\;\mu m^{3}\cdot s^{-1}$ for the flow and $10^{4}\;\mu m\cdot s^{-1}$ for the velocity. This was further supported by detailed distribution profiles of the flow values (see Figure 4C,E) and the velocity values (see Figure 4D,F) for the capillaries and large vessels, respectively. Table A1 and Table A2 illustrate the validation flow and velocity.

### 3.4. The Architecture of the Vascular System Affects the Astrocytic Activity

**Figure 5:**
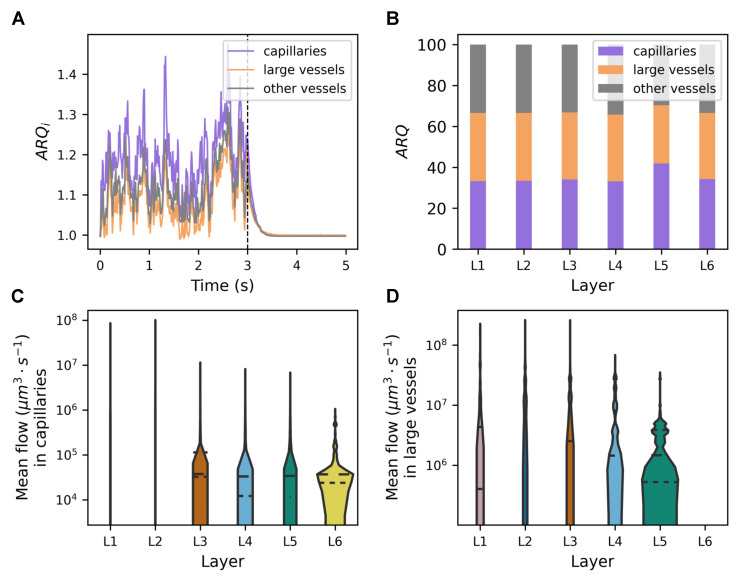
Layer-specific and localized astrocytic activity. (**A**) The average resting state flow ratio, $ARQ_{i}$ from Equation (7), during a five-second period, with the astrocytic stimulation occurring in the first three seconds. The average taken over large vessels is illustrated in orange, that for capillaries is in purple, and that for other vessels is in gray. (**B**) The percentage contribution of the average resting state flow ratio, ARQ from Equation (8), attributed to each vessel type averaged over the initial three seconds of the stimulation. Each bar corresponds to a cortical layer, with orange representing variation due to large vessels, purple due to capillaries, and gray due to other vessels. (**C**,**D**) Violin plots presenting the distribution of the mean flow over time across the six cortical layers in capillaries (**C**) and in large vessels (**D**). Each violin corresponds to a specific cortical layer, and the height of the plot reflects the range of blood flow values within that layer. The width represents the number of segments in that range.

In Figure 5, we compare the extent of flow changes performed by capillaries versus large vessels. Interestingly, the total volume of the capillaries was $1.24\times 10^{7}\;\mu m^{3}$, which occupies 35% of the vascular surface, while the volume of the large vessels was $1.0\times 10^{7}\;\mu m^{3}$, which occupies 28% of the vascular volume. As expected, Figure 5A shows that the average flow ratios in all types of vessels exhibited a similar order of magnitude. The smaller vessels had a higher average flow ratio in the resting state. Figure 5B examines instead the distribution of the flow ratio between layers. The three types of vessels appeared to have the same proportion (one third) across all layers except L5. In general, capillaries, large vessels, and other vessels constituted 35.05%, 31.95%, and 33.00% of the total flow ratio distribution of the resting state, respectively.

Figure 5C,D present violin plots of the distribution of the mean flow over time across the six cortical layers in the capillaries (C) and in the large vessels (D). Each violin corresponds to a specific cortical layer. The height of the plot reflects the range of values of blood flow within that layer, while the width reflects the number of segments in that range. Layer-specific values were more dispersed in L1 and L2. This may have been due to the lower density of capillaries in L1 and L2, coupled with a higher prevalence of larger vessels. Although astrocytic activity affects mainly capillaries, larger vessels such as arterioles and venules are still subject to astrocytic modulation [15,75,76], although to a lesser extent. The impact of astrocytes was represented by the maximum radius deformation. The simulation values were taken from Table 2 and Table 3. The larger diameter and distinct regulatory mechanisms of these vessels attenuated, but did not negate, the impact of astrocytic activity. Capillaries, in particular, are highly responsive to astrocytic signaling, with changes in astrocytic activity that directly influence their diameter and therefore affect the dynamics of blood flow [5].

### 3.5. Impact of Astrocytic Activity in Blood Flow Is Spatially Localized

**Figure 6:**
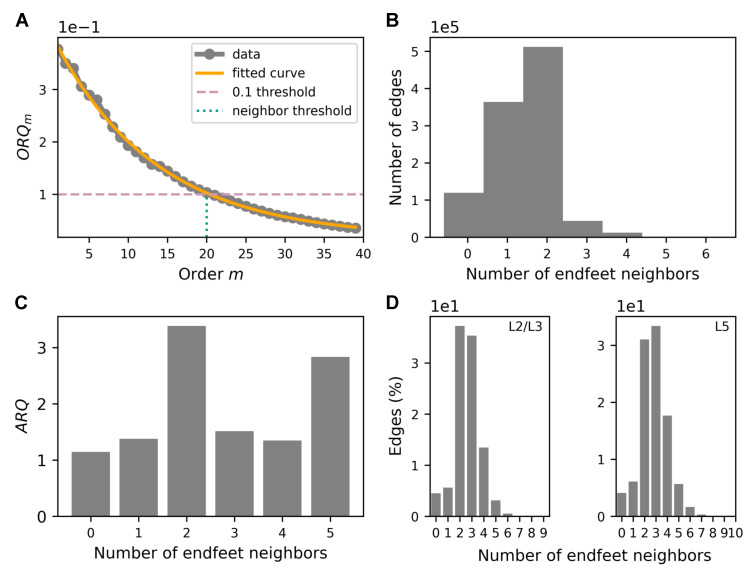
The effect of astrocytic activity on the local vasculature. (**A**) The order flow ratio, $ORQ_{m}$ from Equation (9), of the change in the flow between edges linked to an endfoot and their adjacent edges (see Section 2.7). The x-axis shows the order, *m*, defined as the number of segments starting from the one linked to an endfoot. Data are shown in gray (dotted line), the 0.1 threshold is indicated in pink (dashed line), and the neighbor threshold (order of *m* = 20) is depicted in blue–green (dotted line). The data can be fitted accurately by an exponential function (orange line): $f\left(m\right)=a\;e^{-b\;m}+c$. The final fitting parameters are a=0.39, b=0.07, and c=0.01. (**B**) The distribution of the number of edges based on their proximity to the endfeet. The x-axis indicates the number of endfeet neighbors (close to each edge), while the y-axis represents the number of edges. (**C**) The variation in the flow concerning the nearest endfeet. The *x*-axis represents the number of closest endfeet, while the y-axis illustrates the average resting state flow ratio, ARQ, defined in Equation (8). (**D**) The distribution of the absolute flow variation with respect to the nearest endfeet. The x-axis denotes the number of nearest endfeet related to each edge above a 0.1 threshold (see (**A**)), while the percentage of edges is depicted on the y-axis. The left panel illustrates the distribution of flow across L2/L3, while the right panel displays the distribution of flow across L5.

To explore the effect of astrocytic activity on the local vasculature, we looked at astrocyte-induced flow variation in neighboring segments. In Figure 6, we investigate the spatial range in which a given endfoot had an effect. To that end, we computed the ratio of the resting flow variation between a segment containing an endfoot and its neighbors of increasing order. We defined the order number of the segment, *m*, from an endfoot segment, *k*, the nth segment from both directions (see Section Order Ratios). The order ratio defined in Equation (9) is illustrated in Figure 6A by a gray dotted line. The ratio decreased exponentially with order, indicating that the further a segment was from the endfoot, the less impact the endfoot activity had on the segment flow variation. Section 4 analyzed this effect further. We then chose a 0.1 threshold, below which the average order flow ratio was very low and considered negligible. We then introduced an upper bound limit, m=20, focusing only on the edges connected to endfeet up to m=20 (see Figure 6A).

For each edge in the vasculature, we performed the following steps:

- Take an edge, *k*, in the vasculature;
- Consider all neighbors up to an order of 20 (see Section Order Ratios);
- Count the number of endfeet in the neighbors;
- Classify the edge, *k*, into five categories depending on the number of endfeet (from zero to five, which is the maximum value we found) (see Figure 6B);
- Consider the resting state ratio for the flow at each time for the edge, *k*, and take the mean;
- Consider all edges, *k*, that belong to the same category and take the mean (see the mean flow variation due to astrocytic activity (Figure 6C)).

We observed that segments of the vasculature were more likely to have one or two effective neighboring endfeet. Surprisingly, we found that the highest flow variation occurred when segments had two neighboring endfeet, rather than more (with the exception of the group of five neighboring endfeet). This unexpected result suggests that when the astrocytes had more than two neighboring endfeets, their effects could sometimes have canceled out each other out, leading to a lower impact on flow variation (see Figure 6C). The high flow variation value for the group with five neighboring endfeet may have been due in part to the relatively low number of segments in that group. Finally, we examined the distribution of the seven groups of segments in layers L2 and L3 on one hand and L5 (Figure 6D) on the other hand. The majority of edges in both L2/L3 and L5 had between two and three endfeet neighbors. In other words, the radii of the majority of edges were influenced by two or three endfeet. This was fortuitous, as this two-neighboring-endfeet configuration coincided with the most effective configuration in terms of astrocytic control of blood flow variation (Figure 6C). Although the two-neighboring-endfeet configuration was slightly more likely in subgranular layers, while the three-neighboring endfeet configuration was slightly more likely in supragranular layers, both configurations exhibited similar characteristics across all layers.

## 4. Discussion

### 4.1. Model Presentation

We characterize a crucial mechanism of blood flow regulation in the brain: vasodilation of blood vessels due to astrocytic stimulation. We chose rat SSCx as the foundation of our studies because of its extensive coverage in the literature. The model consisted of several elements:

1. A piece of reconstructed vascular network from the rat SSCx that spanned several cortical layers (L1–L5 completely and L6 partially [18]);
2. Astrocytes carefully placed to populate the space surrounding the vasculature, as well as their endfeet to complete the coupling with the vascular network [8];
3. A set of differential equations (see Section 2) coupling the astrocyte activity (i.e., the radius dilation caused by the endfeet) and the resulting variation in the blood flow values.

### 4.2. Astrocytic Activity Affects Flow in the Vascular Network

We selected the ROU process to model the dynamics of vessel radius changes due to astrocytic activity. To accurately characterize flow variations in response to radius changes, we defined new metrics: resting-state ratios. These metrics were applied to both the radius and the blood flow (see Section 2). Experimental data on radius ratios for vessel segments exclusively linked to endfeet are sparse [14,41,43,44,45,60,61,62] because measuring radius changes requires simultaneous measurements in several vessels. We used these data from the literature as constraints for our model to set the maximum vasodilation for capillaries (38%) and large vessels (23%). This constraint induced our simulations to produce a near-unity flow ratio in most segments connected to endfeet, indicating a high degree of stability in the flow rates even during the initial astrocytic activity period. The distribution of radius ratios in Figure 3A follows a smooth trend, indicating that the changes in the diameter of the vessel occurred predictably over time. In contrast, the distribution of flow ratios in Figure 3B appears scattered, highlighting a highly nonlinear relationship between the changes in the radius of the vessel and the resulting flow variations. The evolution of radius and flow averaged across all vessel segments revealed transient increases during stimulation, followed by a return to baseline levels in the passive phase (see Figure 3D–F). The vasodilation-induced mean flow increase was possibly mediated by astrocytes, consistent with their established role in the regulation of cerebral blood flow. Together, these results imply that astrocytes help stabilize flow dynamics, ensuring consistent cerebral blood flow. In turn, this ensures a reliable supply of oxygen and nutrients to neurons [2,66], prevents ischemic conditions, and supports metabolic homeostasis [2,77,78].

### 4.3. Physiological Evidence Supports Astrocyte–Vasculature Interactions

Our mathematical model of cerebral blood flow focuses on the interactions between astrocytes and the vasculature, providing a detailed framework for understanding their role in the regulation of blood flow. The model predicts astrocyte-induced changes in vascular tone, a mechanism supported by experimental findings that highlight how astrocytic calcium signaling drives arteriole dilation and modulates blood flow [79]. This correspondence emphasizes the physiological validity of our results. Furthermore, the model highlights how localized astrocytic activity affects vascular perfusion and flow heterogeneity. These findings align with in vivo studies that demonstrate astrocyte-mediated vascular responses to metabolic demands [68]. Our findings align with experimental evidence showing the role of astrocytes in maintaining vascular homeostasis and regulating blood flow under normal conditions [80]. While the model does not address pathological conditions, such as acute stroke or reperfusion injury, it lays a robust theoretical foundation for exploring these dynamics in future research. Our study captures the spatial and functional heterogeneity of vascular responses. Our approach could then deepen our knowledge of how astrocytes contribute to blood flow regulation in mammalian physiology.

### 4.4. Simulation Results Reproduced Flow Ranges in the Literature

We validate our model simulations by addressing two key aspects:

1. The comparison of flow values in representative vessels (capillaries and large vessels, Figure 4A) to biological measurements;
2. The response of flow to time-dependent vasodilation induced by astrocytic activity (Figure 4C–E).

Quantifying flow in a dense and interconnected microvascular network like the rat SSCx is experimentally challenging [46]. Although our simulations produced higher flow values than those reported experimentally, several factors could explain this. First, the simulations may have captured physiological phenomena that were difficult to measure directly. Second, flow measurement techniques can introduce artifacts; for example, detection devices may consume a small portion of the flow, affecting accuracy. Third, the sample size differences were substantial: our model simulated numerous segments, whereas experimental studies typically analyze fewer than 50 segments [72,73,74,81,82,83,84,85]. Lastly, our model did not account for the biphasic nature of flow due to red blood cells (RBCs). For computational simplicity, we assumed a fixed RBC volume [86] and hematocrit proportion [87], which may not have captured the effects of RBC compression in capillaries, where smaller diameters can alter RBC volume and flow dynamics. Modeling this compression and incorporating vasoconstriction would likely yield a more symmetric and physiologically accurate flow distribution [88]. Indeed, our model only accounted for vasodilation and disregarded vasoconstriction. If we modeled both vasodilation and vasoconstriction, we might have expected the flow distribution to be more symmetric around the resting state ratio. This is because vasoconstriction would balance the effects of vasodilation, potentially smoothing out the extremes and leading to a more even distribution of flow values. The distribution of flow and velocity values could show additional peaks corresponding to the points where vasoconstriction counteracts vasodilation (see Figure 4C–F). However, this does not necessarily mean that it would be smoother; rather, it would be more balanced, reflecting the bidirectional modulation of the vessel radii. The overall shape could become more complex, indicating the interplay between constrictive and dilative forces in flow dynamics. Schmid et al. demonstrated the impact of RBCs on microvascular perfusion and highlighted the heterogeneity of changes in the distribution of RBCs [88].

It is crucial to acknowledge these potential sources of discrepancy when interpreting the disparities between the simulated and the literature values. By comparing the simulation results (see Figure 4A,B) with the literature data [14,15,16,17,89], we showed that the mean values of the simulated flow and velocity closely aligned with the reported values in the literature, with the majority of data falling within the typical range observed for rats. Indeed, the ranges from the model included the ranges in the literature and could be reproduced by the model.

### 4.5. Global Architecture of Vasculature Affects Astrocytic Activity

The simulations showed that the highest variability in blood flow (67%) occurred inside the capillaries. This underscores the pivotal role of capillaries in fine-tuning the dynamics of cerebral microvasculature flow [14]. In addition, this aligns with the widely accepted notion that capillaries are key sites for modulating blood flow to meet the metabolic demands of neural tissue. The morphology of the vasculature led to different patterns of the distribution of the flow specific to the layer. Deeper layers (L4–L5–L6), with a higher capillary contribution, exhibited narrower flow value ranges, indicating tighter regulation (Figure 5A–D, [34,38,88,90]). In contrast, the upper layers, abundant in larger vessels, showed greater flow fluctuations, likely influenced by capillary dilation [25,91,92].

### 4.6. Impact of Astrocytic Activity on Blood Flow Is Spatially Localized

We introduced the $ORQ_{m}$ metric (Equation (9)) to quantify astrocytic activty effects on the local vasculature. We observed a reduction in this ratio with an increasing segment order (distance). It may be attributed to the dispersion of the perturbation caused by the branching of the segments (see Figure 6A). This analysis showed that astrocytes have localized effects on nearby blood vessels (that is, on the first 20 neighbors considering an arbitrary threshold of 10% Figure 6A).

Despite their widespread presence, a comprehensive understanding of endfeet’s physiology is still lacking. This knowledge gap is in part due to the limited identification and characterization of the specific proteins that differentiate the endfoot from the rest of the astrocyte body. Our findings suggested a relationship between astrocytic coverage and blood flow regulation where moderate astrocytic presence (two or three effective endfeet) generates a robust response, while excessive coverage (more than three endfeet) leads to diminished effects (see Figure 6C,D). This observation is supported by the literature [93]. More precisely, the interaction between multiple neighboring endfeet can present diminishing returns. Instead, the collective influence of multiple neighboring endfeet can lead to complex interactions, constructive or destructive interference, and heightened flow variability in certain scenarios. Finally, if endfeet are spatially clustered or arranged in a specific pattern, such a setup can contribute to higher flow variability. Layer-specific configurations that influence flow regulation highlight the intricate interplay between cellular components and vascular function in cortical regions. Layer-specific endfoot configurations further modulate flow, with supragranular layers optimized for two endfeet and subgranular layers for three [94,95].

Although it was assumed in our model that capillary diameters could change dynamically with activity, it is important to note that this remains a subject of debate in the literature. Evidence suggests that the first vessels of a penetrating arteriole can exhibit rapid dilation [14], while capillaries in the middle of the network tend to show slower or negligible dilation on the scale of seconds and respond only significantly over longer periods, such as hundreds of seconds [96,97].

### 4.7. Perspectives and Future Directions

Our current modeling approach provides a robust framework for understanding astrocyte-mediated regulation of vessel diameters. However, there are several promising directions to expand its applicability. Future iterations could incorporate experimental validation through in vivo imaging techniques and manipulations of astrocytic activity. Such experiments would directly test the predictions of the model, particularly concerning localized flow changes and capillary perfusion dynamics. Expanding the model to include a complete brain vasculature dataset, such as the mouse brain vasculature by Ji et al. [98], would enable more comprehensive simulations of regional variations in flow regulation. This would involve repairing vasculature gaps and classifying vessel types using anatomical references like the Xiong et al. [99]. Furthermore, exploring the application of the model in pathological conditions, such as neurodegenerative diseases or stroke, could provide insight into altered blood flow mechanisms and potential therapeutic targets. Integrating other cellular components, such as pericytes, represents another compelling direction. Pericytes are known to dynamically modulate capillary flow through contractile activity and signaling mechanisms along the endothelium [100]. By incorporating their dynamics and upstream endothelial signaling, future models could simulate more realistic neurovascular interactions and provide a deeper understanding of blood flow regulation across cortical layers.

### 4.8. Limitations

While our model effectively simulates astrocyte-driven vasodilation and its effects on cerebral microcirculation, it relies on simplifying assumptions and parameters derived from experimental data. Therefore, there are still many avenues for improvement that have yet to be explored. For instance, the model assumes fixed red blood cell (RBC) volumes and hematocrit proportions, neglecting the effects of RBC compression within capillaries. This simplification may overlook significant contributions to flow dynamics, as shown by studies on the deformation and distribution of RBCs [86,88]. Additionally, the model focuses solely on vasodilation, disregarding the role of vasoconstriction. Incorporating bidirectional vessel modulation would likely produce a more physiologically accurate representation of flow dynamics, as vasoconstriction counterbalances vasodilation to stabilize flow distributions.

We acknowledge that the neurovascular unit is highly complex and includes additional mechanisms of blood flow regulation that our model does not explicitly address. For instance, recent studies have shown that pericytes, positioned at capillary junctions, can dynamically modulate blood flow through contractile activity and signaling mechanisms that propagate along the endothelium [100]. These findings highlight the importance of pericyte-mediated hemodynamic control, which our model does not incorporate. Furthermore, ion channel activity in pericytes, including inward-rectifier channels $K^{+}$ and voltage-dependent channels, has been shown to modulate capillary hemodynamics, further contributing to the regulation of cerebral blood flow [101]. Despite sharing molecular machinery with smooth muscle cells, pericytes exhibit distinctive ion channel properties that influence cerebral blood flow differently. Although these mechanisms are integral to the functioning of the neurovascular unit, our model does not explicitly incorporate pericyte dynamics, as the focus remains on the astrocyte-mediated regulation of vessel diameters. Future iterations of our model should aim to incorporate upward signal transfer within the endothelium, as well as the integration of pericyte and endothelial signaling to better represent the effects of the neurovascular unit’s network and simulate more realistic flow dynamics. Incorporating these network effects would allow for a more detailed analysis of how local changes propagate and influence the overall flow distribution across the cortical layers. By acknowledging these limitations and areas for improvement, our study lays the foundations for more sophisticated models that include pericyte dynamics, endothelial signaling, and bidirectional vessel modulation, which would provide a more comprehensive understanding of neurovascular coupling.

Despite alignment with anatomical observations, our approach represents a simplification compared to more advanced hemodynamic models that account for distributed inflows in finer anatomical details. Future research could explore the incorporation of these features, such as the shear-dependent flow distribution and spatially distributed inflows. Including these features would enhance the fidelity of the model, but would require significant computational resources and detailed parameterization. Despite these limitations, the current model serves as a foundation for future advancements that can integrate these complex mechanisms into a unified framework for neurovascular research.

## 5. Conclusions

In conclusion, this study sheds light on the intricate interplay between astrocytic signaling and cerebral blood flow dynamics. We provide a useful framework for future research into physiological and pathological conditions associated with neurovascular dysfunctions.

## Figures and Tables

**Table 1:** Vasculature network parameters. Volumetric analysis of the cortical layers and vascular system. The experimental data of the vessel volumes within each cortical layer. CAP stands for capillaries, while LV stands for large vessels. The low numbers within L6 in all columns can be attributed to the limitations of the experimental dataset. The flow ratio is the proportion of flow inside capillaries and large vessels. The values do not add up to 100% because we did not consider middle-size vessels.

Layer	Layer Volume (μm^3^)	Vascular System Volume (μm^3^)	Volume Occupied by Vessels (%)	Flow Ratio (%)	
				CAP	LV
L1	1.29 × 10^8^	5.03 × 10^6^	3.88	66.17	3.27
L2	1.16 × 10^8^	1.16 × 10^6^	4.24	67.25	2.64
L3	2.77 × 10^8^	9.39 × 10^6^	3.40	62.98	2.76
L4	1.48 × 10^8^	4.35 × 10^6^	2.92	26.61	1.28
L5	4.12 × 10^8^	8.25 × 10^6^	2.00	35.46	1.33
L6	5.49 × 10^8^	3.47	0.06	53.15	0

**Table 2:** Simulation parameters in capillaries.

Number of Time Steps	Time Step Size	Time to Peak	Maximum Radius Ratio
500	0.01 s	2.7 s *	1.38 *

Values marked with an asterisk (*) are based on data in the literature [14,41,43,44,45,60,61,62].

**Table 3:** Simulation parameters in large vessels.

Number of Time Steps	Time Step Size	Time to Peak	Maximum Radius Ratio
500	0.01 s	3.3 s *	1.23 *

Values marked with an asterisk (*) are based on data in the literature [14,41,43,44,45,60,61,62].

**Table 4:** The refined validation of the simulation outputs. The flow values in capillaries are grouped according to their diameter.

Diameter (μm)	Number of Edges	Flow in Capillaries ($\mu m^{3}\cdot s^{-1}$)		
		min	Max	Mean
1–2	7106	0.01	2.22 × $10^{5}$	6.95 × 10^3^
2–3	21,140	0.11	2.72 × 10^6^	3.03 × 10^4^
3–4	14,228	1.60	5.52 × 10^6^	1.60 × 10^5^
4–5	90,748	7.74	9.05 × 10^6^	4.40 × 10^5^
5–6	59,670	0.79	1.89 × 10^7^	9.10 × 10^5^
6–7	24,853	8.83	2.14 × 10^7^	1.48 × 10^6^

**Figure A1:**
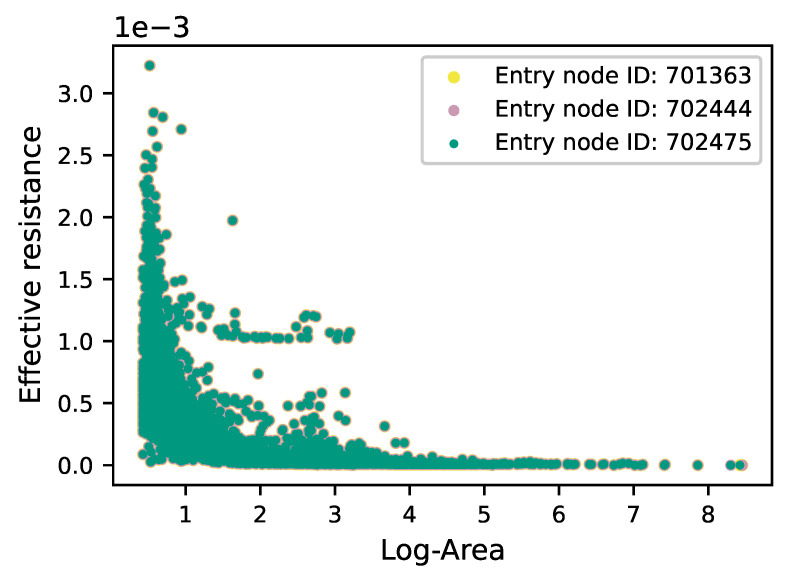
Effective resistance. A scatter plot depicting the effective resistance (ER) as a function of the logarithm of the exit node area. This analysis involves the calculation of effective resistance between each of the three selected entry nodes and all exit nodes in the network.

**Figure A2:**
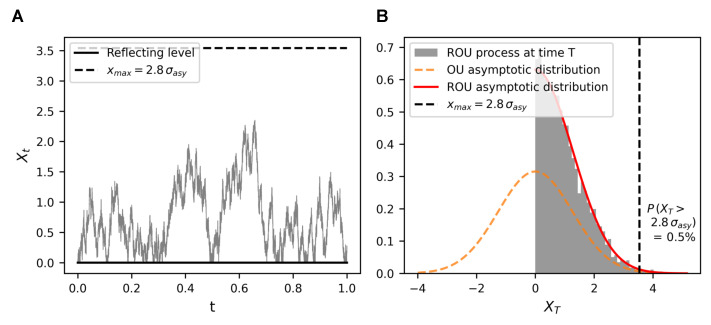
The calibration of the Ornstein–Uhlenbeck (OU) process. (**A**) The sample path of a reflected OU (ROU) process with T=1, κ=5, and σ=4. The solid black line represents the reflecting model, while the dashed black line denotes a realistic maximum value for the ROU process. (**B**) A comparison of the asymptotic OU and ROU distributions. We simulated 5000 ROU paths and constructed a histogram using the values of $X_{T}$ at T=1. The solid red line represents the ROU asymptotic distribution, the dashed red line represents the OU asymptotic distribution, and the dashed black line denotes a realistic maximum value for the ROU process.

**Figure A3:**
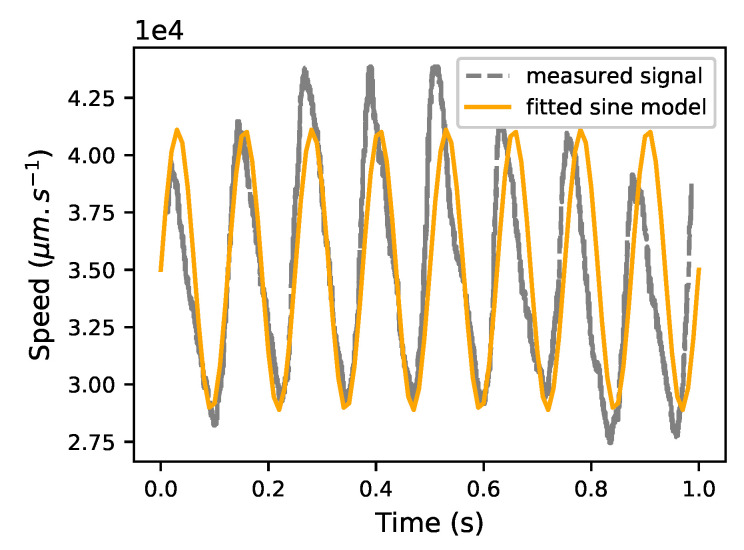
Fit of a sinusoidal blood flow signal. Comparison of blood velocity signal (gray dashed line) obtained from [103], with fitted sine function v(t)=Asin(2πft)+C (orange continuous line) with $A=6119\;\mu m\cdot s^{-1}$, $f=8\;s^{-1}$, and $C=35,000\;\mu m\cdot s^{-1}$.

**Figure A4:**
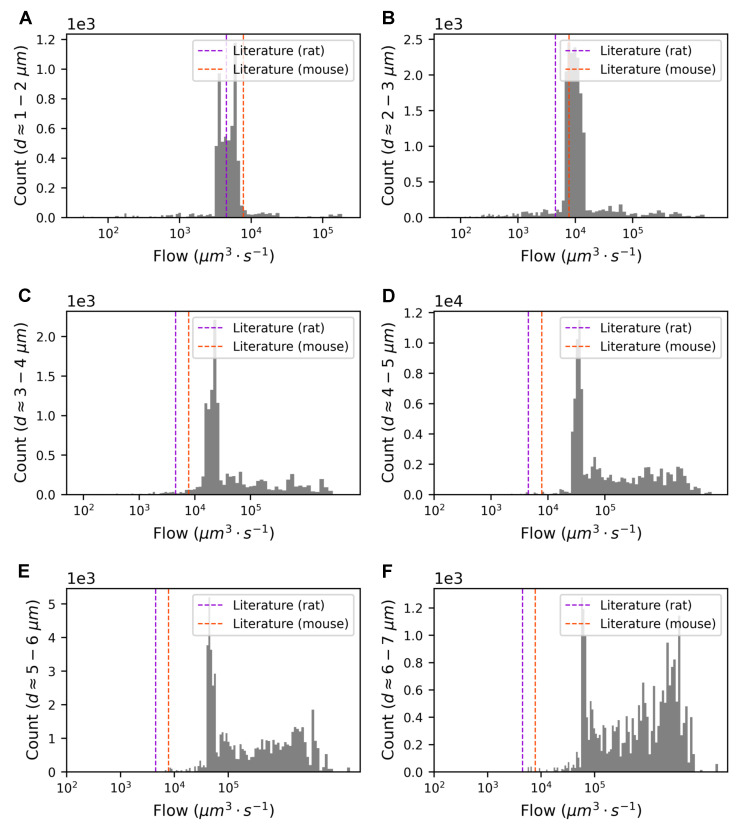
Validation of simulation results against literature data. Distribution of blood flow in capillaries categorized by diameter plotted on logarithmic scale, with dashed lines from previous studies (see Appendix B). (**A**) 1–2 μm, (**B**) 2–3 μm, (**C**) 3–4 μm, (**D**) 4–5 μm, (**E**) 5–6 μm, and (**F**) 6–7 μm.

**Figure A6:**
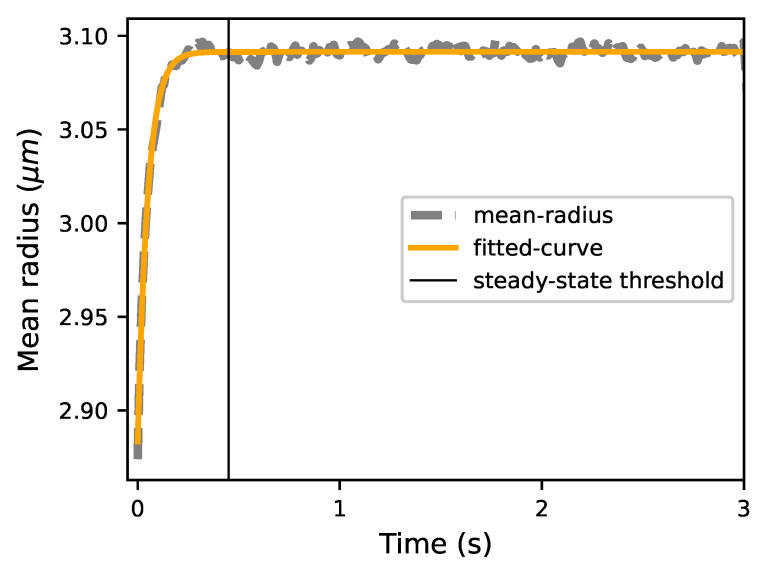
The dynamical evolution of the average radii. A comparison of the average radii over time (gray dashed line), with a fitted exponential function $f\left(t\right)=ae^{-b\;t}+c$ (orange continuous line) with the fitted parameters a=−0.23, b=21.38, and c=3.03.

## Data Availability

The algorithm, implemented in Python, was released as an open-source package named AstroVascPy. The source code is available for public access on GitHub [55]. The software was designed for scalability across diverse vascular network datasets, with the possibility to use parallel computation with modern HPC clusters.

## Appendix A

### Appendix A.1. Mathematical Framework of Blood Flow Model

The resistance in Equation (2) was a modification introduced in [46] of the classical Poiseuille resistance to account for the influence of hematocrit. We used the same values of the model parameters α=0.863, β=14.3μm, γ=27.5, and δ=0.351μm provided in [46].

Making the further assumption that the speed was constant with respect to time and space, i.e., $\frac{\partial u(t,x)}{\partial t}=0$ and $\frac{\partial u(t,x)}{\partial x}=0$, it follows that Equation (3) becomes the well-known Hagen–Poiseuille equation:

(A1) $$\frac{dp(x)}{dx}=-R\left(r\right)u.$$

Let us consider a single edge between node *i* and node *j*. We denoted with $R_{ij}$ the resistance, R(r), associated with the selected edge and with $A_{ij}$ the section area. Let us integrate both sides of the equation between the positions $x_{i}$ and $x_{j}$ of the two nodes:

(A2) $$\begin{array}{cc} p_{j}-p_{i} & =-R_{ij}\;l_{ij}\;u_{ij}\\ \; & =-\frac{R_{ij}\;l_{ij}}{A_{ij}}\;Q_{ij}. \end{array}$$

The term $u_{ij}$ indicates the velocity between *i* and *j*, $l_{ij}$ is the length of the edge, and $Q_{ij}=u_{ij}A_{ij}$ is the flux (also called flow in the next sections). Let us recall

(A3) $${\tilde{R}}_{ij}=\frac{R_{ij}\;l_{ij}}{A_{ij}},$$

such that

(A4) $$p_{i}-p_{j}={\tilde{R}}_{ij}\;Q_{ij}.$$

We consider the following flux conservation condition and replace the expression of $Q_{ij}$:

(A5) $$\sum_{j∼i}Q_{ij}\;=\;\sum_{j∼i}\frac{p_{i}-p_{j}}{{\tilde{R}}_{ij}}\;=\;0$$

where the sum is over the node *j* connected to node *i*. It is convenient to rewrite our equations in matrix–vector notation. Let us introduce the N×M-oriented model. incidence matrix *B* (with *N* nodes and *M* edges) defined as $B_{i,k}=1$ and $B_{j,k}=-1$, with *k* representing the index of the oriented edge, connecting nodes *i* to node *j*. The signs indicate the orientation of the graph edges. We define the graph Laplacian *L* as

(A6) $$\begin{array}{c} L\;=BW^{-1}B^{T}, \end{array}$$

where *W* is the M×M diagonal matrix of edge resistances (i.e., $W_{kk}={\tilde{R}}_{ij}$). We rewrite Equation (A5) into matrix–vector notation, including the boundary conditions, as follows:

(A7) $$\begin{array}{c} L\;p=Q^{b}\;, \end{array}$$

where p is the unknown pressure vector and $Q^{b}$ is a given vector containing the boundary flows on the nodes of degree one, with values equal to zero on the internal nodes with a degree greater than one. The vector $Q^{b}=\left(Q_{1}^{b},Q_{2}^{b},...\right)$ satisfies the property $\sum_{i=1}^{N}Q_{i}^{b}=0$, corresponding to the vanishing total flow condition.

Once the pressure is computed, we can retrieve the flux vector since Equation (A4) can be written in vector form as follows:

(A8) $$\begin{array}{c} Q=W^{-1}B^{T}p\;. \end{array}$$

Often, boundary flow is approximated using various methods, such as building a larger homogeneous vasculature with simple boundary conditions [71].

Appendix A.2 explains how we computed the boundary flows. Once they were set, the solution of Equation (A7) could be computed up to a constant pressure value. It is common practice to set it equal to the external pressure: 10 mmHg (1.33 g·μm^−1^·s^−2^) [102].

### Appendix A.2. Blood Flow on the Boundary Nodes

First, we injected the flow into the largest $\tilde{n}$ nodes (with the greatest diameter) located in the top region of the vasculature. This choice was consistent with the actual location and direction of the artery measured in the rat SSCx [103]. In particular, we defined the top as the zone where $y>y_{min}+0.95\;\left(y_{max}-y_{min}\right)$ was *y* the vertical coordinate of a cortical column oriented with the lowest layers on top (L1), down to the lowest layer (L6) at the bottom. This was consistent with the actual location and direction of the artery measured in rat SSCx [103].

After that, we needed to determine the amount of flow to inject. Kim et al. [103] provide the time series of blood velocity for large vessels. Figure A3 shows that the velocity signal had a sinusoidal shape. It was convenient to fit the empirical velocity signal with a sine wave:

(A9) $$v\left(t\right)=\tilde{A}\;\sin\left(2\pi f\;t\right)+C,$$

where $\tilde{A}\in R$ is the sine amplitude, f∈N is the sine frequency, and C∈R is the signal baseline. Taking into account a unit interval for the time, t∈[0,1], such that the sine domain is a multiple of a 2π period, the parameters were estimated as follows: *C* is the mean value of the signal, $\tilde{A}$ is $\sqrt{2}$ times the standard deviation of the signal, and *f* is the number of peaks. Following this approach, the fitted parameters were as follows: $\tilde{A}=6119\;\mu m\cdot s^{-1}$, $f=8\;s^{-1}$, and $C=35,000\;\mu m\cdot s^{-1}$.

Once we knew the velocity, v(t), we computed the flow to inject into the entry node, *i*:

(A10) $$Q_{i}\left(t\right)=A_{i}\left(t\right)\cdot v\left(t\right),$$

where $A_{i}\left(t\right)$ is the area of the entry node, *i*, at the time *t*.

The last step was to determine the flow in the exit nodes. Suppose that there were $\tilde{m}$ exit nodes. We defined the weight, $w_{j}\left(t\right)$, as follows:

(A11) $$w_{j}\left(t\right)=\frac{A_{j}\left(t\right)}{A_{tot}\left(t\right)}\;\mathrm{with}\;A_{tot}\left(t\right)=\sum_{j=1}^{\tilde{m}}A_{j}\left(t\right),\;1\leq j\leq \tilde{m}.$$

From the total flux conservation law, we know that the total exit flow, $Q_{tot}^{out}\left(t\right)=\sum_{j=1}^{\tilde{m}}Q_{j}\left(t\right)$, and the total entry flow, $Q_{tot}^{in}\left(t\right)=\sum_{i=1}^{\tilde{n}}Q_{i}\left(t\right)$, must satisfy

$$Q_{tot}^{in}\left(t\right)=-Q_{tot}^{out}\left(t\right)$$

at each time t∈[0,T].

Thus, the flow in the exit node *j* is as follows:

(A12) $$Q_{j}\left(t\right)=-w_{j}\left(t\right)\;Q_{tot}^{in}\left(t\right).$$

Appendix A.5 provides a detailed overview of the history of exit flow computation, leading to the current version discussed above.

### Appendix A.3. Stochastic Endfoot Activity

A generic ROU process, $X=\{X_{t}:0\leq t\leq T\}$, is described by the following stochastic differential equation:

(A13) $$dX_{t}=-\kappa X_{t}dt+\sigma dW_{t}\;+dL_{t},\;\;\mathrm{with}\;\;X_{0}=0,$$

where κ is the reversion-to-zero coefficient and σ is the diffusion coefficient.

(A14) $$\begin{array}{cc} W=\{W_{t}:0\leq t\leq T\}, & \;\;\mathrm{is}\;a\;\mathrm{Wiener}\;\mathrm{process},\\ L=\{L_{t}:0\leq t\leq T\}, & \mathrm{is}\;\mathrm{the}\;\mathrm{minimal}\;\mathrm{non}-\mathrm{decreasing}\;\mathrm{process}\\ \;\;\mathrm{that}\;\mathrm{ensures}\;X_{t}\geq 0\;\mathrm{for}\;\mathrm{all}\;t\geq 0,\\ \;\;\mathrm{and}\;\mathrm{is}\;\mathrm{also}\;\mathrm{known}\;\mathrm{as}\;\mathrm{the}\;\mathrm{local}\;\mathrm{time}. \end{array}$$

The simulation time [0,T] is discretized in time steps I∈N of size Δt. Let us indicate with $X_{i}$ the value of *X* in the time step i≤I. $X_{i}$ can be calculated using the following recursive relation:

(A15) $$X_{i+1}=\max\left\{e^{-\kappa \Delta t}X_{i}+\tilde{\sigma }\epsilon_{i}\;,\;0\right\}$$

where $\tilde{\sigma }=\sqrt{\frac{\sigma^{2}}{2\kappa }\left(1-e^{-2\kappa \Delta t}\right)}$ and $\epsilon_{i}∼N\left(0,1\right)$ is a standard normal random variable.

The radius dynamics $r_{i}$ for 0≤i≤I can be modeled as

(A16) $$r_{i}=r_{0}+X_{i}.$$

where $r_{0}$ is the reference resting state radius.

### Appendix A.4. Reflected Ornstein–Uhlenbeck Process Calibration

The ROU parameters κ and σ are calibrated from the values of the maximal radius extension $r_{max}$ and the mean time $t_{max}$ to reach it from $r_{0}$. It is useful to recall that the asymptotic distribution of the ROU process follows the half-normal law:

(A17) $$f\left(x;\;\kappa ,\sigma \right)=\frac{2}{\sigma }\sqrt{\frac{\kappa }{\pi }}\;e^{-\frac{\kappa }{\sigma^{2}}\;x^{2}}\;\;\mathrm{for}\;\;x\geq 0.$$

In the region x≥0, this distribution is exactly twice the asymptotic distribution of the OU model, which is a normal distribution with a mean of 0 and a standard deviation of $\sigma_{asy}=\sqrt{\frac{\sigma^{2}}{2\kappa }}$.

For convenience, we decided to measure the distance from the origin as a multiple of $\sigma_{asy}$. Let us introduce the new parameter c>0 and set

(A18) $$x_{max}=c\;\sigma_{asy}.$$

Our main modeling choice is to set c=2.8. With this assumption, we want to introduce a realistic maximum value for the ROU process. The probability $P(X_{T}>x_{max})\approx 0.5\%$ is small enough to be ignored. Larger values of *c* would make it difficult for the process to reach $x_{max}$. In Figure A2 we can see a sample path and a histogram of the ROU process with the new introduced maximum value.

In the calibration process, we can retrieve the value of $x_{max}$ from the experimental values of $r_{max}$ and $r_{0}$ by setting $x_{max}=r_{max}-r_{0}$. Equation (A18) expresses the parameter σ in terms of κ:

(A19) $$\sigma \left(\kappa \right)=x_{max}\frac{\sqrt{2\kappa }}{c}.$$

The expected time, u(x), to reach $x_{max}$ for an ROU process starting from *x* is the solution of the following differential equation (see [53]):

(A20) $$-\kappa x\frac{du(x)}{dx}+\frac{1}{2}\sigma^{2}\frac{d^{2}u\left(x\right)}{dx^{2}}=-1$$

with $u^{\prime }\left(0\right)=0$ and $u(x_{max})=0$. This boundary value problem has mixed boundary conditions. We numerically integrate this second-order ordinary differential equation employing a finite difference scheme using a backward difference for the first-order derivative and a central difference for the second-order derivative.

The expected time, starting from x=0, obtained from Equation (A20), depends only on the parameter κ since σ can be substituted from Equation (A19). For this reason, we indicate this dependence as $u_{\kappa }\left(0\right)$:

(A21) $$u_{\kappa }\left(0\right)-t_{max}=0.$$

This equation can be solved numerically for κ using a root finding algorithm, such as the Brent method [104].

### Appendix A.5. Exit Flow Computation

Our current approach to calculate the flow at the exit nodes (presented in Appendix A.2) is a naive approach that simply distributes the total entry flow among the exit nodes proportionally to their respective area.

Here, we present an alternative approach that we considered at the beginning of our analysis but which we later decided to discard because it required too many computational resources. In this section, we consider weights proportional to the effective conductance $c_{ab}$ between an entry node, *a*, and an exit node, *b*:

(A22) $$w_{ab}\;\propto \;c_{ab}\;:=\;\frac{1}{r_{ab}}$$

The effective conductance is the inverse of the effective resistance. Interestingly, this is a popular distance measure used in graph theory and electrical networks [105].

If we associate each node, *i*, with an element, $e_{i}$, of the standard basis of the Euclidean vector space, $R^{n}$, we can define the effective resistance between any nodes, *i* and *j*, as follows:

(A23) $$\begin{array}{c} r_{ij}={\left(e_{i}-e_{j}\right)}^{T}L^{†}\left(e_{i}-e_{j}\right) \end{array}$$

where $L^{†}$ is the pseudo-inverse of the graph Laplacian matrix. The Laplacian matrix is a singular matrix and, therefore, we need to consider the pseudo-inverse.

The weight definition proposed in this section is more appropriate as it considers the overall effective conductance between an entry node and an exit node, which depends on the length and radius of each edge within the graph, rather than solely relying on the area of the exit node.

Our graph contains $10^{6}$ nodes, out of which 19,004 are boundary nodes. As we chose three entry nodes, the remaining 19,001 are exit nodes. Given these values, even when employing optimized parallel algorithms in distributed systems, explicitly calculating the pseudoinverse of the Laplacian matrix becomes exceedingly time consuming.

Consequently, we opted to compute solely the effective resistance between the entry node, *a*, and the exit node, *b*, ignoring the values of $r_{ij}$ between nodes that we were not interested in. If we call $v_{ab}:=e_{a}-e_{b}$, we can efficiently solve the linear system $Lx=v_{ab}$ to obtain $x=L^{†}\;v_{ab}$ and then obtain $r_{ab}=v_{ab}^{T}\;x=x\left[a\right]-x\left[b\right]$.

Although this approach is considerably faster than calculating the pseudoinverse, it remains highly time-consuming, as it requires the solution of 3 × 19,001 linear systems and cannot be used in practice. Due to this constraint, we opt for the simpler area-based approach, which nonetheless yields comparable results.

As depicted in Figure A1, we observe that the effective resistance exhibits a decreasing relationship with the area. Although this functional relationship is not linear, indicating that the two approaches yield very different weights, this coarse analysis provides evidence that a greater area corresponds to an increased effective conductance. Thus, we employ this heuristic argument to justify our chosen approach.

## Appendix B. Model Validation

The validation focused solely on the blood vessels in the internal part of the vasculature, within the defined bounds of [$x_{min}$, $x_{max}$], [$y_{min}$, $y_{max}$], and [$z_{min}$, $z_{max}$] with $x_{min}=67.70\;\mu m$, $x_{max}=622.30\;\mu m$, $y_{min}=1033.50\;\mu m$, $y_{max}=1684.99\;\mu m$, $z_{min}=273.27\;\mu m$, and $z_{max}=926.73\;\mu m$. So, approximately 17% of the vasculature fell within the defined region. In this way, we minimized the possible distortions introduced by the boundary conditions and take into account the contributions of all nodes in the vasculature.

## Appendix C. Choice of Time Step

To select an appropriate time step for our simulation, we considered the time evolution of the mean radius in the vasculature, as shown in Figure A6. The objective was to choose a time step that was not too large. We observed that the slope of the curve almost flat after time t≈0.4. The region where the curve is almost flat is usually called the steady state, or plateau. Considering a time step size of Δt=0.01, we estimate that approximately 40 time steps were required to reach the plateau (see Figure A6).

The shape of the curve representing the average radius evolution during stimulation can be described by the following parametric function:

(A24) $$f\left(t\right)=ae^{-b\;t}+c.$$

## Appendix D. Passive Properties of Network

The passive properties of the network refer to the intrinsic characteristics of the vascular network. The intrinsic characteristics are responsible for the dynamics of the radii and flow inside the vasculature when astrocytic activity ends. This is what we call passive activity (that is, the passive response of the vasculature). In our model, the phase corresponding to the passive response of the vasculature can be described by Equation (A13) by setting σ=0.

By defining τ as the time at which the stimulation ceases, after setting σ=0 in Equation (A13), we obtain the subsequent dynamics:

(A25) $$X_{t}=X_{\tau }e^{-\kappa (t-\tau )}\;\mathrm{for}\;t>\tau .$$

Here, $X_{t}$ represents the radius of the blood vessel at time *t*, $X_{\tau }$ is the radius of the blood vessel at the cessation of stimulation τ, and κ governs the rate of decay. This equation describes the exponential decay of the blood vessel radius after the cessation of astrocytic stimulation.

The average radius can be determined through

(A26) $${\bar{R}}_{t}=e^{-\kappa (t-\tau )}\;\frac{1}{N}\;\sum_{i=1}^{N}R_{\tau }^{i}\;\mathrm{for}\;t>\tau .$$

Here, ${\bar{R}}_{t}$ represents the average radius of the blood vessel at the time *t* after the cessation of stimulation. The sum $\sum_{i=1}^{N}R_{\tau }^{i}$ calculates the initial radii of *N* blood vessels $segment_{v}essel_{o}ver_{t}imes$$R_{\tau }^{i}$ at the time of cessation of stimulation. The exponential decay factor accounts for the decrease in the radius of the blood vessel over time, reflecting the continued influence of the withdrawal of astrocytic activity. These formulas show how blood vessel radius evolves following the cessation of astrocytic stimulation.

**Table A1:** The validation of the simulation results against data from the literature. The units of flow values are expressed in $\mu m^{3}\cdot s^{-1}$ (see [72], we cite as * in the table).

	Flow in Capillaries		Flow in Large Vessels	
	Average	Range	Average	Range
The literature	6.65 × 10^3^	4.85 × 10^3^–7.78 × 10^3^	-	1 × 10^5^ – 1 × 10^7^ *
Our network	4.93 × 10^5^	0.01–1.83 × 10^7^	2.28 × 10^7^	1.54 × 10^5^–1.59 × 10^8^

**Table A2:** The validation of the simulation results against data from the literature. The units of velocity values are expressed in $\mu m\cdot s^{-1}$. **: [73,74,81,82,83,84,85], *: [72].

	Velocity in Capillaries		Velocity in Large Vessels	
	Average	Range	Average	Range
The literature	1.370 × 10^3^ **	4 × 10^2^–2 × 10^3^ **	-	2 × 10^3^–3 × 10^4^ *
Our network	2.47 × 10^4^	3 × 10^3^–8.6 × 10^5^	6.26 × 10^4^	2.73 × 10^2^–3.69 × 10^5^

**Table A3:** Unit conversions and parameters for blood flow calculations.

Parameter	Value/Conversion
Flow rate in large vessels	$\mathrm{nl}\cdot s^{-1}$
Flow rate in capillaries	$\mathrm{RBC}\cdot s^{-1}$
Velocity	$\mathrm{mm}\cdot s^{-1}$
Millimeter to micrometer	$1\;\mathrm{mm}=10^{3}\;\mu m$
Femtoliter to cubic micrometer	$1\;fL=1\;\mu m^{3}$
RBC volume conversion	$\frac{\mathrm{RBCv}}{\mathrm{hematocrit}}\cdot \mathrm{RBC}$
RBC volume (RBCv)	56.51fL [86]
Hematocrit proportion	0.45 [87]

RBCv indicates the volume of a single red blood cell. Baskurt et al. [86] reported a RBCv of 56.51 fl in rats. According to Pries et al. [87], the hematocrit proportion is 0.45.
